# Supplementary material for: Topological superfluid defects with discrete point group symmetries
Source: Nat Commun. 2022 Aug 8;13:4635. doi: 10.1038/s41467-022-32362-5 (PMC9360439; doi:10.1038/s41467-022-32362-5)
Supplement: Supplementary file 1 — Supplementary Information [file 41467_2022_32362_MOESM1_ESM.pdf]

Supplementary Information for

Topological Superfluid Defects with Discrete Point Group Symmetries

Y. Xiao, M.O. Borgh, A.A. Blinova, T. Ollikainen, J. Ruostekoski, and D.S. Hall

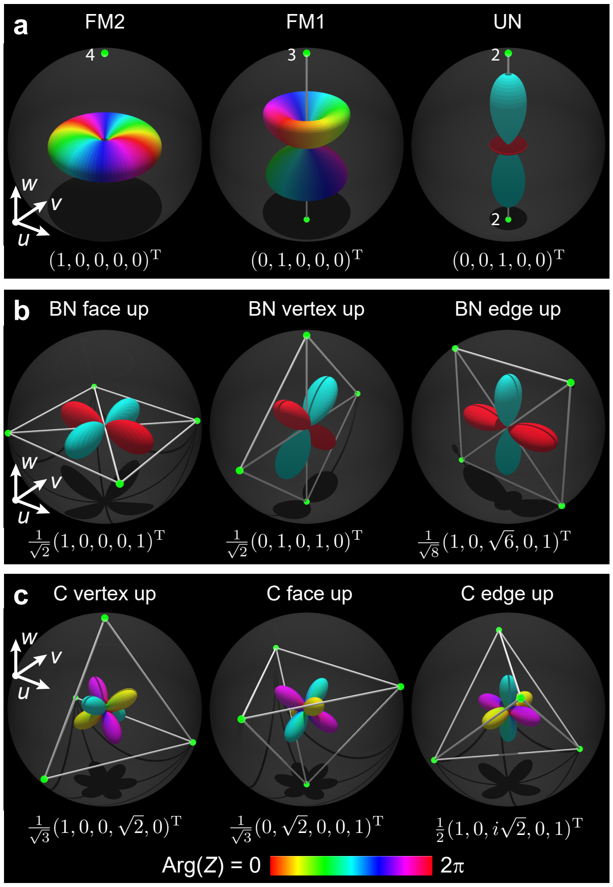

Supplementary Figure 1 | Magnetic phase symmetries of spin-2 Bose–Einstein condensates. **a** Prototype spinors, Majorana and spherical harmonics representations of the magnetic phases without discrete polytope symmetry: ferromagnetic-2 (FM2), ferromagnetic-1 (FM1), and uniaxial nematic (UN). **b** The representations of the biaxial nematic (BN) phase with the Majorana symmetry of a square, for three different orientations. **c** The representations of the cyclic (C) phase with the Majorana symmetry of a tetrahedron, for three different orientations. The Majorana points are shown as green dots with adjacent numbers indicating multiplicities > 1.

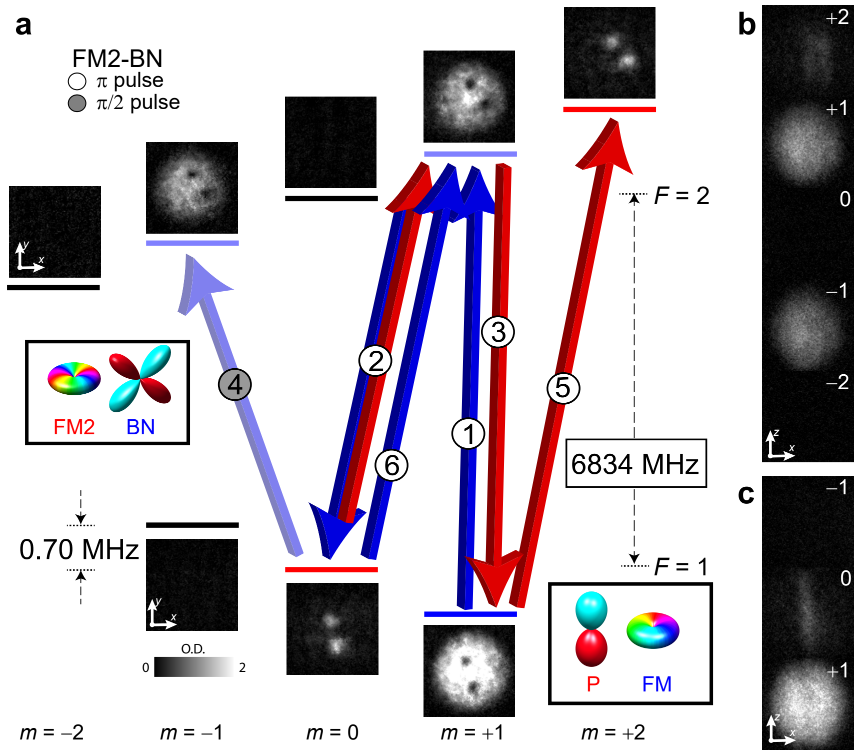

Supplementary Figure 2 | Creation of singular vortices in the biaxial nematic (BN) phase with ferromagnetic-2 (FM2) cores. **a** The circled numbers give the order of the microwave transitions within the hyperfine levels (solid lines), beginning with a vortex in the spin-1 ferromagnetic (FM) phase with polar (P) core in the $|F, m\rangle$ states shown. The red arrows indicate the path of the cores through the internal states of the system, and the blue arrows indicate the path of the vortices. In the fourth pulse (pale blue arrow), the $\pi/2$ rotation angle transfers half of the population from the $|F = 1, m = 0\rangle$ spinor component to the $|2, +1\rangle$ component. The experimental images show column densities taken from **a** the top, and the side for **b** $F = 1$ and **c** $F = 2$, expressed in units of optical depth (O.D.) with a field of view of 212 μm × 212 μm. The images from the upper and lower hyperfine levels are from different condensates.

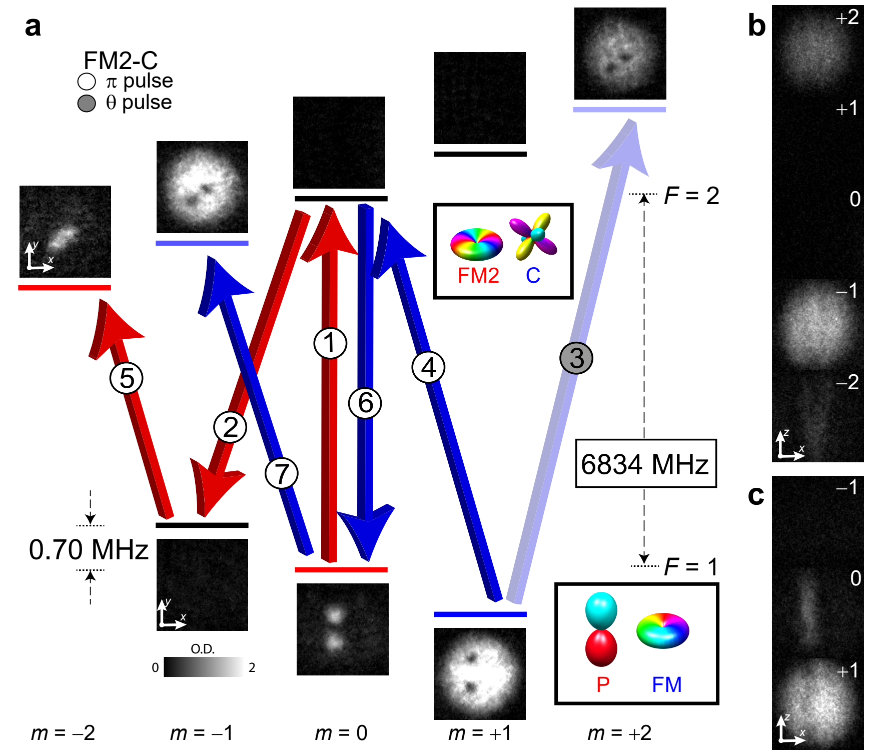

Supplementary Figure 3 | Creation of singular vortices in the cyclic (C) phase with ferromagnetic-2 (FM2) cores. **a** The thick lines schematically show the hyperfine levels ($F = 1$ and $F = 2$, with Zeeman levels m reading left to right) in a magnetic field of 1 G, accompanied by experimental images of the condensate viewed along the z-axis after Stern–Gerlach separation. The connecting arrows illustrate the pulse sequence, with order given by the circled number, colored blue for transitions involving the components with phase singularities and red involving the superfluid components filling those singularities. The sequence begins with a vortex in the spin-1 ferromagnetic (FM) phase with polar (P) core. In the third pulse (pale blue arrow), the rotation angle $\theta = 2\arcsin(1/\sqrt{3})$ transfers 1/3 of the population from the $|F = 1, m = +1\rangle$ spinor component to the $|2, +2\rangle$ component. The experimental images show column densities taken from **a** the top, and the side for **b** $F = 1$ and **c** $F = 2$, expressed in units of optical depth (O.D.) with a field of view of 212 μm × 212 μm. The images from the upper and lower hyperfine levels are from different condensates.

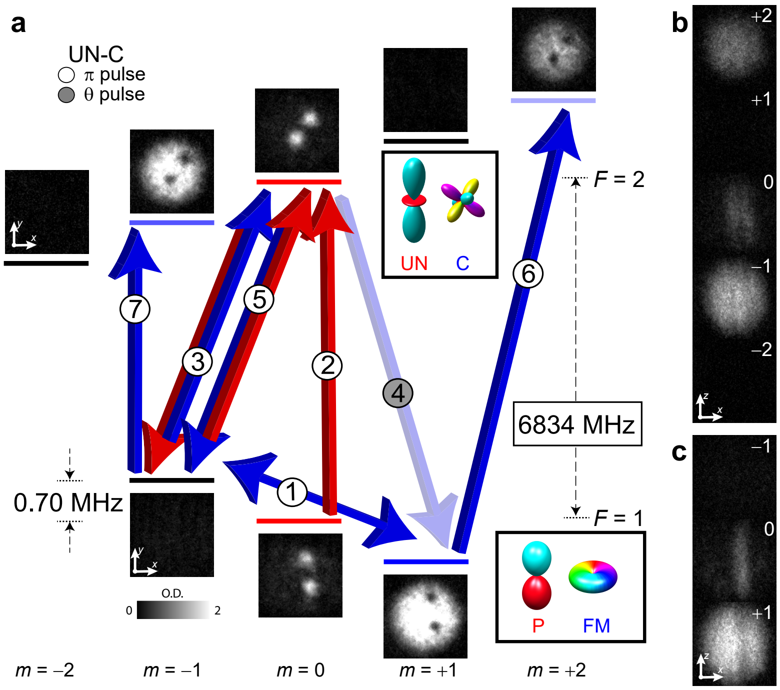

Supplementary Figure 4 | Creation of singular vortices in the cyclic (C) phase with uniaxial nematic (UN) cores. **a** The thick lines schematically show the hyperfine levels ($F = 1$ and $F = 2$, with Zeeman levels m reading left to right) in a magnetic field of 1 G, accompanied by experimental images of the condensate viewed along the z-axis after Stern–Gerlach separation. The connecting arrows illustrate the pulse sequence, with order given by the circled number, colored blue for transitions involving the components with phase singularities and red involving the superfluid components filling those singularities. The sequence begins with a vortex in the spin-1 ferromagnetic (FM) phase with polar (P) core. The first radio-frequency π-pulse converts the $|F = 1, m = +1\rangle$ spinor component into the $|1, -1\rangle$ component, leaving the $m = 0$ component unchanged; a similar sequence, omitting this rotation, can create the face-up C vortex with UN core. In the fourth pulse (pale blue arrow), the rotation angle $\theta = 2\arcsin(1/\sqrt{3})$ transfers 1/3 of the population from the $|2, 0\rangle$ spinor component to the $|1, +1\rangle$ component. The experimental images show column densities taken from **a** the top, and the side for **b** $F = 1$ and **c** $F = 2$, expressed in units of optical depth (O.D.) with a field of view of 212 μm $\times$ 212 μm. The images from the upper and lower hyperfine levels are from different condensates.

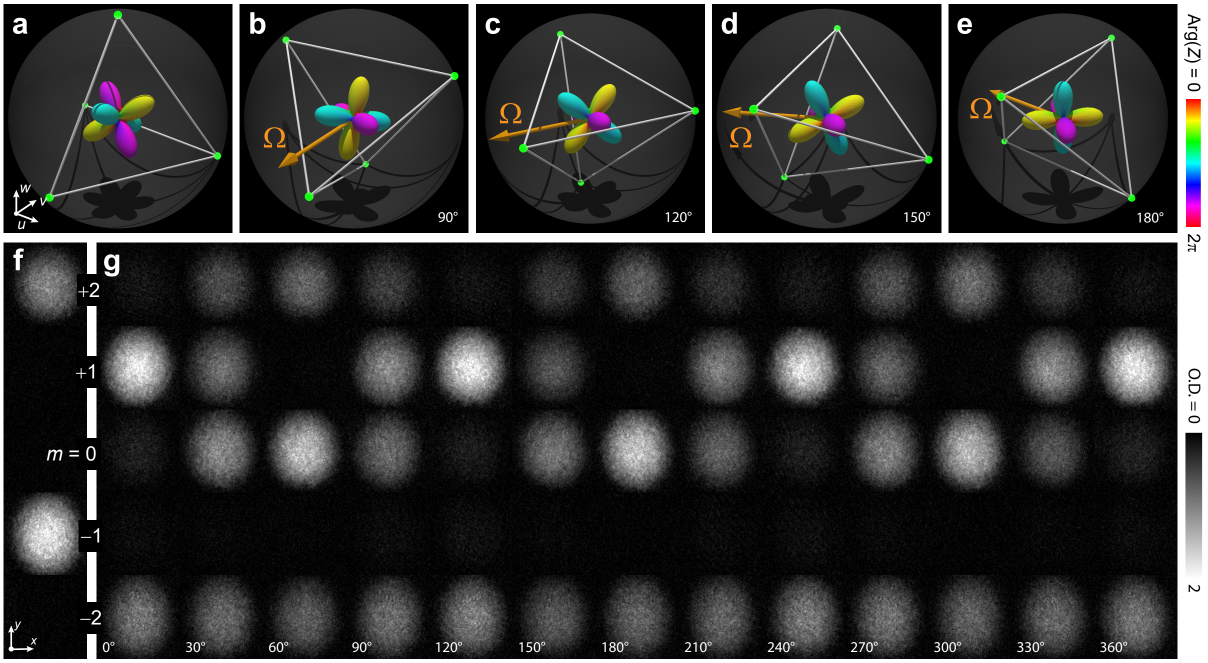

Supplementary Figure 5 | Demonstration of polytope discrete symmetry in the continuous wave function of the spin-2 superfluid in the cyclic (C) phase. The different orientations of the order parameter are obtained by rotations of the spinor with radio-frequency (rf) pulses at different phase angles, as shown schematically by the orange torque vector $\mathbf{\Omega}$. **a** Majorana and spherical harmonics representations of the initial C magnetic phase. **b–e** The rotated C phase after tetrahedral angle rf pulses with the phases 90°, 120°, 150°, and 180°, as shown. **f** Experimental images of the spinor components for the initial magnetic phase. **g** Experimental images of the rotated spinor components, taken from the side after $\pi/2$ rotations at the indicated rf phases. Each experimental subpanel shows a spinor component column density taken from the side in terms of optical depth (O.D.) with a field of view of 219 μm $\times$ 219 μm.
